# Large Middle Cranial Fossa Schwannoma: A Rare Presentation of Vestibular Schwannoma

**DOI:** 10.7759/cureus.33186

**Published:** 2022-12-31

**Authors:** Michael Gelsomino, Akram M Eraky, Ahmed Awad, Youssef Farhat, Saman Shabani, Wade Mueller, Nathan T Zwagerman

**Affiliations:** 1 Neurosurgery, Froedtert Memorial Lutheran Hospital, Medical College of Wisconsin, Milwaukee, USA; 2 Neurological Surgery, Medical College of Wisconsin, Milwaukee, USA; 3 Pathology and Laboratory Medicine, Medical College of Wisconsin, Milwaukee, USA

**Keywords:** antoni structures, subtemporal approach, acoustic neuroma, internal acoustic meatus, internal auditory canal, vestibular cranial nerve, cerebellopontine angle tumour, schwann cells, vestibular schwannoma, middle cranial fossa

## Abstract

Schwannomas are benign tumors composed of neoplastic Schwann cells and rarely occur in the central nervous system. Schwannomas account for approximately 8% of intracranial tumors and most commonly originate from cranial nerve VIII at the cerebellopontine angle in the posterior fossa. Herein, we report two cases of vestibular schwannomas extending in the middle fossa. The first case shows a 51-year-old male who presented with a history of mild headaches for one year associated with acute nausea, vomiting, and word-finding difficulties. Imaging revealed a large multicystic contrast-enhancing lesion in the left middle cranial fossa. The middle fossa lesion was resected with pathology indicating a schwannoma. The second case shows a 63-year-old woman who presented with seizures, right-sided hearing loss, and right-sided facial weakness. On MRI, she is found to have a large right middle fossa lesion originating from the right internal auditory canal and consistent with vestibular schwannoma with a 9 mm leftward midline shift. The histopathologic examination of the excised tumor indicated a schwannoma. Schwannomas most commonly occur in the posterior fossa when they present intracranially. However, in rare occurrences, they may present as middle fossa masses with significant intracranial compression.

## Introduction

Schwannomas (SCs) are benign tumors composed of neoplastic Schwann cells, which do not commonly occur intracranially. SCs account for approximately 8% of intracranial tumors and usually originate from cranial nerve VIII, commonly known as vestibular SC or acoustic neuroma [1]. SCs are typically not large in size and uncommonly occur without association with a cranial nerve [2-4]. Herein, we report two cases of vestibular SC with a rare presentation of large intracranial extension in the middle cranial fossa.

## Case presentation

Case 1

A 51-year-old Caucasian male presented to the neurosurgery service with nausea, emesis, and severe headache. This was preceded by a two-week history of worsening headaches, fatigue, gait instability, and word-finding difficulties. He also had a one-year history of intermittent headaches that were mild and relieved with nonsteroidal anti-inflammatory drugs. He had no other major medical problems, and he had no prior workup for these complaints. On examination, the patient had a mild right-sided weakness with right-sided pronator drift associated with mild difficulty in naming objects. The medical evaluation revealed the presence of multiple bilateral subsegmental pulmonary emboli although no other major medical issues were encountered.

**Figure 1:**
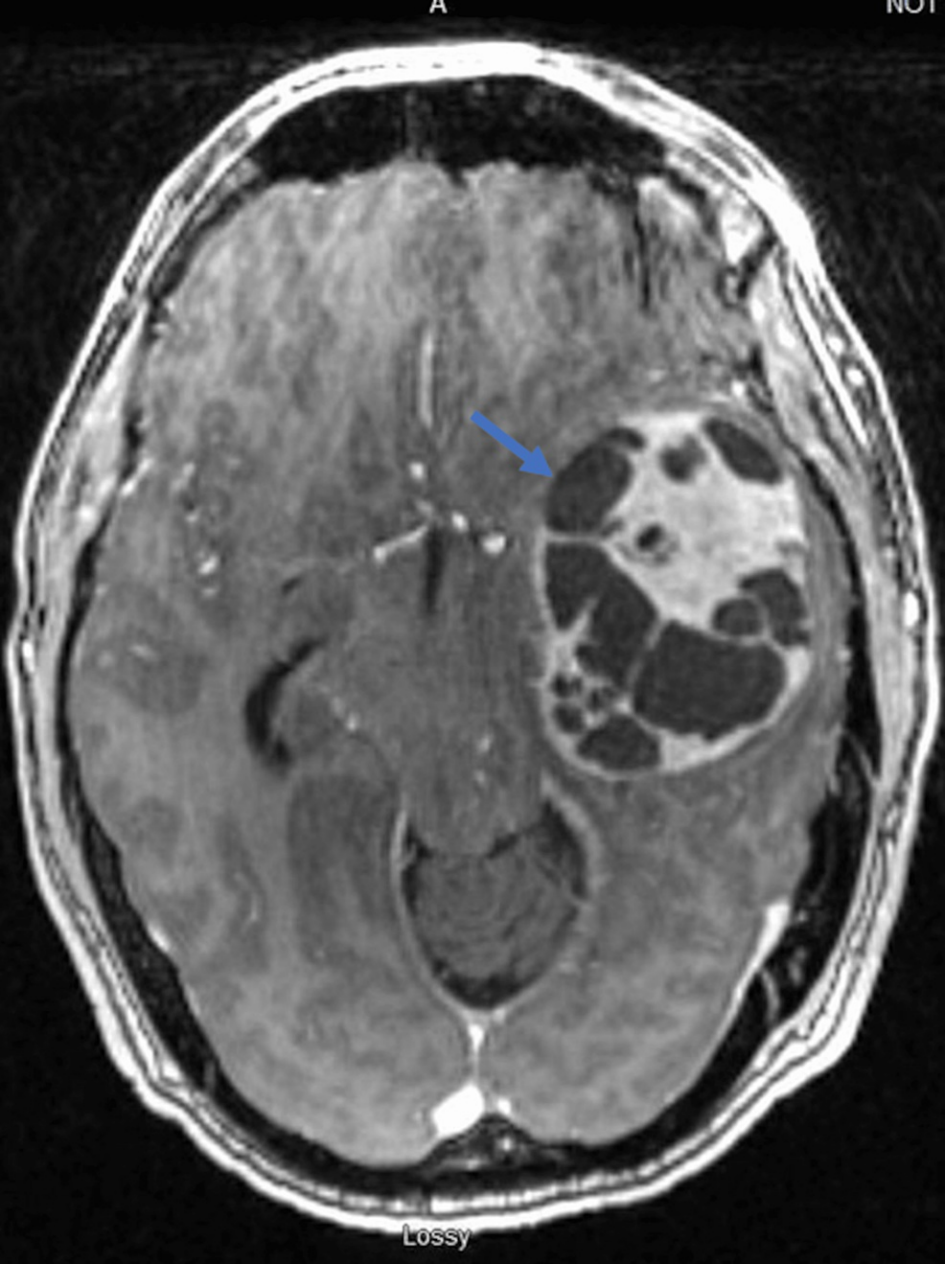
Preoperative MR brain: axial T1 with contrast The view shows avid enhancement of the peripheral portion of the tumor without enhancement of the cystic middle portion.

**Figure 2:**
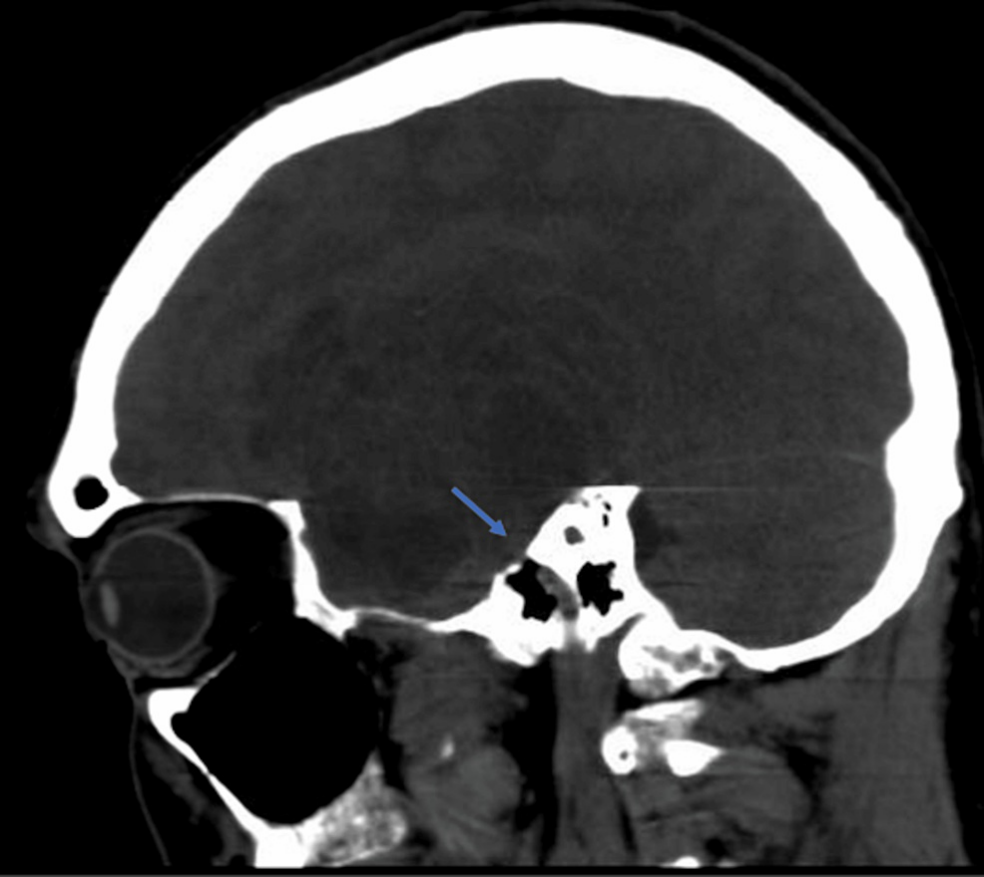
Preoperative CT head: sagittal view without contrast The view shows the mass emanating from the superior aspect of the petrous temporal bone.

A computed tomography (CT) of the head without contrast was obtained, which demonstrated a left-sided supratentorial cystic mass lesion. Further evaluation with contrasted magnetic resonance imaging demonstrated a T1 hypointense lesion measuring 5.0 cm x 6.9 cm x 6.2 cm, which had multiple intralesional cysts. T2-weighted imaging revealed a hypointense lesion with a small amount of perilesional edema. Contrast administration revealed avid enhancement of the peripheral portion of the tumor without enhancement of the cystic middle portion (Figure 1). The mass emanated from the superior aspect of the petrous temporal bone and was in continuity with the internal auditory canal (Figure 2).

**Figure 3:**
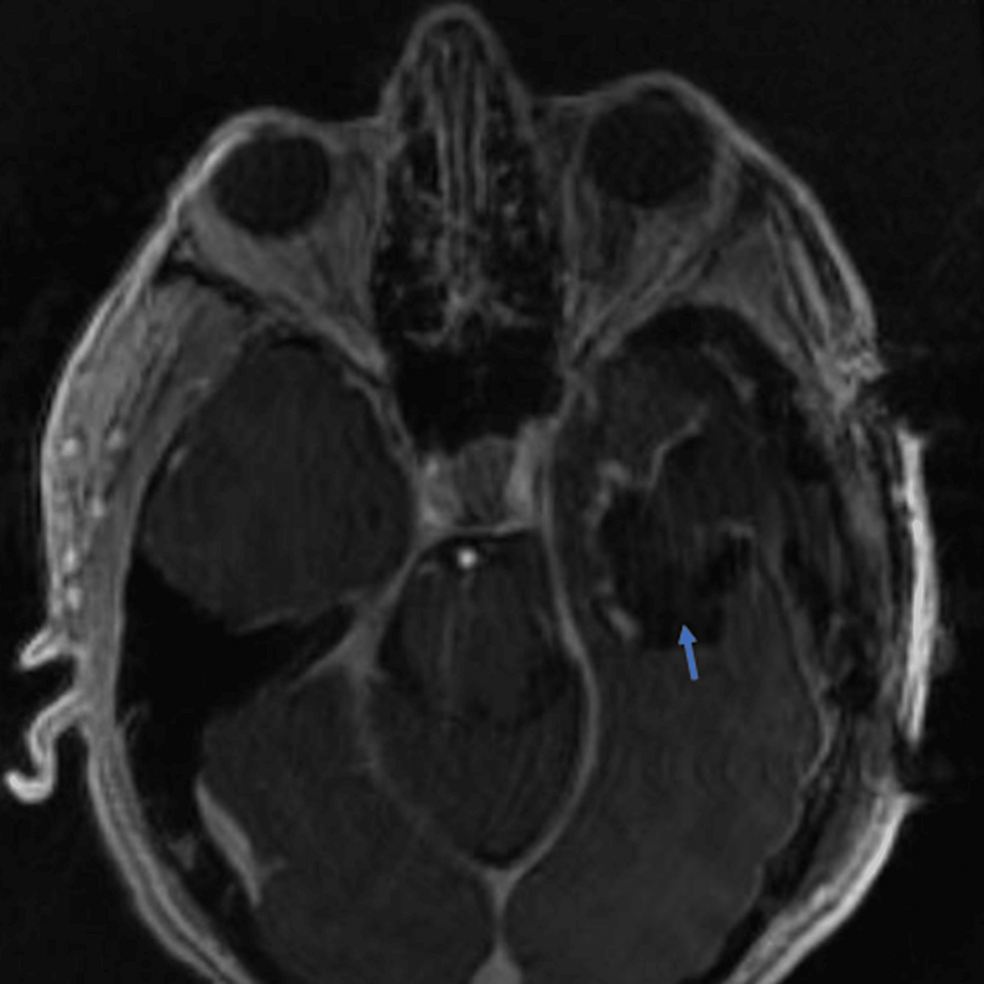
Postoperative MRI: axial T1 with contrast The view shows the gross total resection of the lesion.

An awake left-sided craniotomy and excision of the mass were performed. A subtemporal approach was utilized intraoperatively. A tumor capsule was encountered, and this provided a plane between the mass and brain parenchyma. The patient was tested by a neuropsychologist intraoperatively, and his language function improved during surgery as he was able to name objects better. The middle fossa tumor was resected in its entirety and postoperative MR imaging confirmed that a gross total resection was achieved (Figure 3).

Histologic examination of the mass revealed moderate proliferation of spindle-shaped cells. The architecture of the lesion consisted of spindle cells arranged in fascicular and sheet-like patterns. The nuclei of the cells were oval to spindle-shaped with occasional pleomorphism. The cells stained strongly positive for S-100, vimentin, and SOX-10. Since the size and location of this lesion are rare, it was sent out for an independent analysis. The pathologic diagnosis was independently confirmed by an outside institution, which found that the tissue was consistent with SC.

Postoperatively, the patient recovered well from surgery. His language function continued to improve. Given that pulmonary emboli were found during medical evaluation preoperatively, he was started on anticoagulation several days after surgery and was discharged to home in stable condition.

Case 2

A 63-year-old female patient, who experienced seizures for the first time four years ago, was hospitalized for seizures. She reported right-sided hearing loss and right-sided facial weakness that had started 14 years ago. She was started on steroids and levetiracetam after hospitalization and did not experience seizures since then. Her physical examination was normal except for right-sided facial weakness and right-sided hearing loss. She did not have previous surgeries or major medical comorbidities except for hypothyroidism and hypertension.

**Figure 4:**
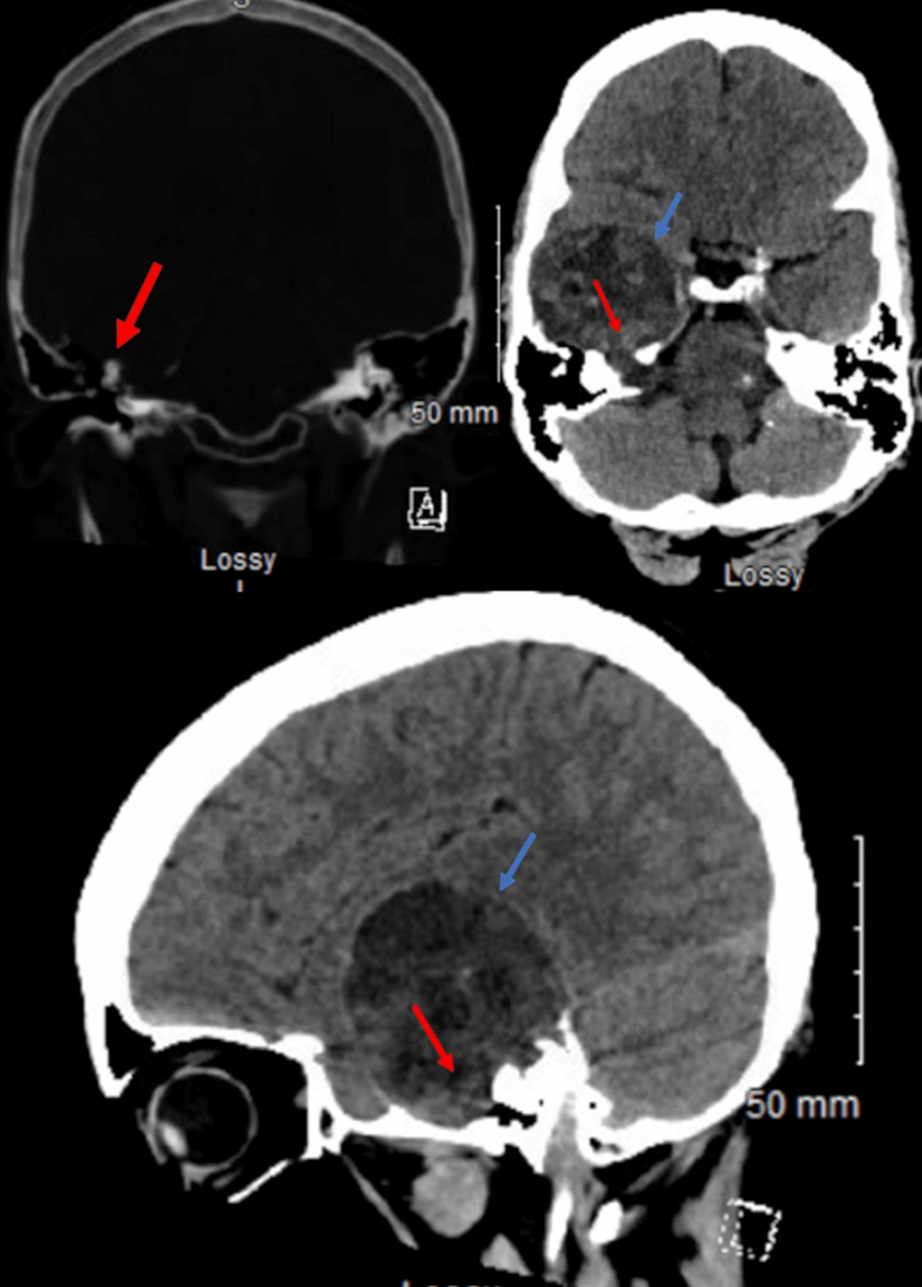
Preoperative CT head: coronal, axial, and sagittal views without contrast The view shows a right 6.2 cm x 5.1 cm x 5.9 cm extra-axial lesion (blue arrow) filling the internal auditory canal and wrapping superiorly and anteriorly across the right temporal bone (red arrow) along the floor of the right middle cranial fossa.

**Figure 5:**
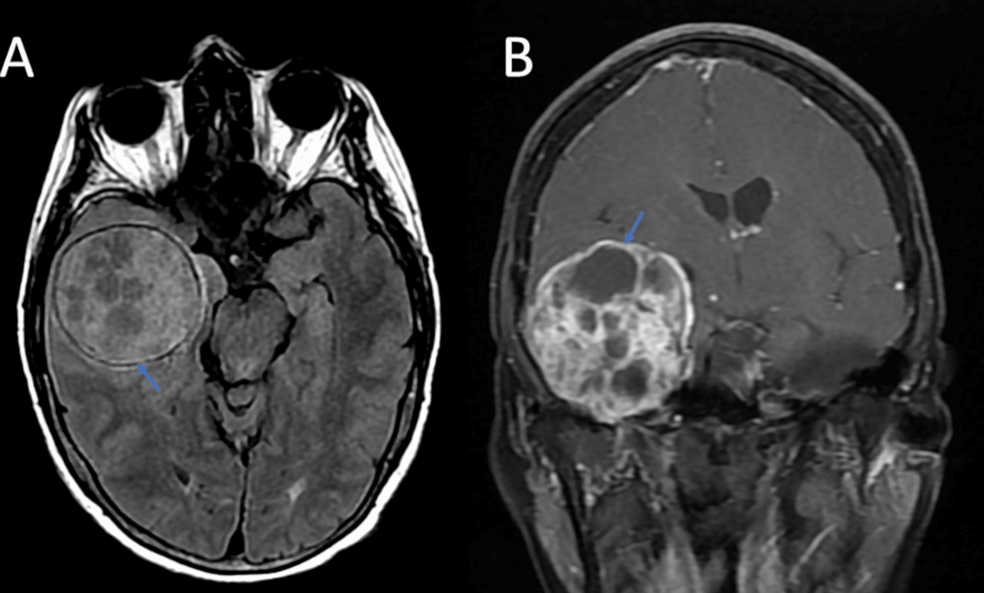
Preoperative MRI: axial T1 without contrast (A) and T1 coronal with contrast (B) Image (A) shows a hypo- to iso-intensity lesion on MRI-T1. Image (B) shows extensive areas of nonenhancement indicating cystic and necrotic changes within the tumor.

On imaging, there was a right 6.2 cm x 5.1 cm x 5.9 cm extra-axial mass filling the internal auditory canal and wrapping superiorly and anteriorly across the right temporal bone, along the floor of the right middle cranial fossa with a 9 mm leftward midline shift (Figure 4). On MRI-T1, it showed hypo- to isointensity lesion, while it showed hyper- to isointensity lesion on MRI-T2 (Figure 5). Extensive areas of nonenhancement indicate cystic and necrotic changes within the tumor (Figure 5).

**Figure 6:**
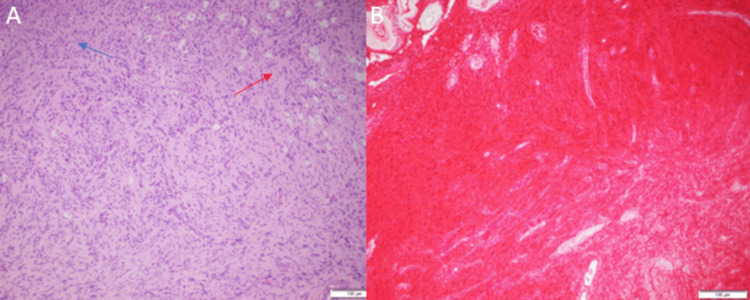
Microscopic examination of the resected lesion stained by hematoxylin and eosin (A) and S-100 immunostain (B) Image (A) shows hypercellular areas called Antoni A (blue arrow) with focal loose reticular areas called Antoni B (red arrow). Also, foamy Schwann cells and structures vaguely resembling Verocay bodies are occasionally seen. Image (B) shows a strongly and diffusely positive immunohistochemical stain for S-100 indicating schwannoma.

She underwent right middle fossa craniotomy and tumor resection. Internal debulking of the tumor took place until a thin rim of the tumor remained; then, this remaining part was separated from the brain parenchyma. There were no complications during the surgery. The microscopic examination of the resected lesion demonstrated a moderately cellular spindle cell tumor arranged in compact fascicular structures (Antoni A architecture) with focal loose reticular areas (Antoni B). Foamy Schwann cells and structures vaguely resembling Verocay bodies were occasionally seen. The blood vessels show marked mural thickening, hyalinization, and hemosiderin pigment deposition (Figure 6). An immunohistochemical stain for S-100 was strongly and diffusely positive (Figure 6). Postoperatively, the patient reported mild right-sided facial numbness that improved a few days later. Moreover, her facial asymmetry was resolved.

## Discussion

SCs are benign tumors originating from Schwann cells, which account for approximately 8% of all intracranial masses [1,3]. Bilateral SCs are associated with neurofibromatosis type two (NF-2) and exist in 95% of NF-2 patients. However, unilateral SC and meningioma can present in NF-2 patients [5]. Theoretically, SCs can originate from any peripheral nerve and any cranial nerve except the optic nerve as it is the only nerve that is not wrapped by Schwann cells but by oligodendrocytes [6]. Intracranial SCs are most commonly associated with cranial nerve VIII and are typically found in the posterior fossa [4,7,8].

In most cases, vestibular SCs grow initially inside the internal auditory canal and then extend into the cerebellopontine angle. In MRI, it is characterized by the absence of a cerebrospinal fluid (CSF) signal in the internal auditory canal [9]. The mass reported in our two cases likely originated from cranial nerve VIII although they were located in the middle cranial fossa as its growth eroded petrous temporal bone. The bony erosion allowed the mass to grow superiorly where it displaced the adjacent brain parenchyma. Cranial nerve VIII is not the only nerve that is associated with supratentorial SCs as lesions in association with the olfactory, trigeminal, and nasoethmoid nerves have been reported as well [7,10-16].

As SCs tend to be slow growing, this mass reached a large size before causing symptoms severe enough that the patient sought care. The location of the mass determines the symptoms [4]. In the first case, the patient had some speech difficulties as the tumor caused a mass effect on the adjacent left temporal lobe, which was the dominant side for this patient. In the second case, the patient complained mainly of seizures, right hearing loss, and right facial weakness due to tumor compression on the seventh nerve, eighth nerve, and temporal lobe.

Although the tumors are typically well-circumscribed, imaging characteristics are largely nonspecific for SCs, and larger tumors may exhibit cystic degeneration as seen in our two cases [17,18]. On MR imaging, SCs present as hypo- to isointense on T1-weighted images and hyper- to hypointense on T2-weighted images [17,19,20]. The mass in our patient demonstrated erosion of the temporal bone and communication with the internal auditory canal, which hinted that it may have originated from cranial nerve VIII. SCs can be found in association with other cranial nerves as well, with the fifth and ninth cranial nerves being the next most common after cranial nerve eight [2].

Histologic analysis is necessary to diagnose such a lesion as meningioma, or other extra-axial masses could present in a similar manner [21]. Meningiomas are S-100 negative, whereas SCs are S-100 positive. Moreover, SCs are also negative for glial fibrillary acidic protein. Histology for SCs also demonstrates Antoni A areas showing high mitotic activity and hypercellularity and Antoni B structures showing deformed blood vessels and hypocellularity [22]. Accurate diagnosis is important as adjuvant therapy is not necessary for SCs, and gross total resection can be curative.

## Conclusions

Intracranial SCs of the middle cranial fossa can grow to be rather large and may present with symptoms that are not commonly seen in association with these masses. The imaging characteristics tend to be nonspecific although clues to a possible diagnosis can be obtained based on the proximity of the lesion to adjacent structures, such as the petrous bone and internal auditory canal, and the symptoms resulting from the vestibular and facial nerve involvement, such as hearing loss and facial paralysis. Gross total resection should be obtained when feasible and could be curative.
